# A potential threat to malaria elimination: extensive deltamethrin and DDT resistance to *Anopheles sinensis* from the malaria-endemic areas in China

**DOI:** 10.1186/1475-2875-12-164

**Published:** 2013-05-17

**Authors:** Duo-quan Wang, Zhi-gui Xia, Shui-sen Zhou, Xiao-nong Zhou, Ru-bo Wang, Qing-feng Zhang

**Affiliations:** 1 National Institute of Parasitic Diseases, Chinese Center for Disease Control and Prevention, WHO Collaborating Centre for Malaria, Schistosomiasis and Filariasis; Key Laboratory of Parasite and Vector Biology, Ministry of Health, Shanghai 200025, People’s Republic of China

**Keywords:** Insecticide resistance, *An*. *sinensis*, Malaria-endemic areas

## Abstract

**Background:**

Insecticide resistance in malaria vectors is a growing concern in many countries and requires immediate attention because of the limited chemical arsenal available for vector control. There is lack of systematic and standard monitoring data of malaria vector resistance in the endemic areas, which is essential for the ambitious goal of malaria elimination programme of China.

**Methods:**

In 2010, eight provinces from different malaria endemic region were selected for study areas. Bioassays were performed on F1 progeny of *Anopheles sinensis* reared from wild-caught females using the standard WHO susceptibility test with diagnostic concentrations of 0.25% deltamethrin and 4% DDT.

**Results:**

For *An*. *sinensis*, the results indicated that exposure to 0.25% deltamethrin of F1 families with mortalities ranging from 5.96% to 64.54% and less than 80% mortality to DDT at the diagnostic concentration of 4% across the study areas.

**Conclusions:**

*Anopheles sinensis* was completely resistant to both deltamethrin and DDT, and resistance to pyrethroid has risen strikingly compared to that recorded during 1990s. The results highlight the importance of longitudinal insecticide resistance monitoring and the urgent need for a better understanding of the status of insecticide resistance in this region.

## Background

The use of pyrethroid insecticides in malaria vector control has increased dramatically in the past decade through the scale up of insecticide treated net distribution programmes and indoor residual spraying campaigns [1]. The ambitious goal of malaria elimination programme of China launched in July 2010 and planned to be completed by 2020. However, malaria is still an important disease in some area. Vector control has played an essential role in the reduction of malaria in the regions and is still indispensable to control malaria in endemic foci [2]. The available vector control methods rely on the use of insecticides for bed net impregnation or indoor spraying. Consequently, development of insecticide resistance may jeopardize the vector control efforts. Hence, knowledge of vector resistance and trends in target areas are basic requirements to guide insecticide use for malaria elimination. With the prevalence and severity of resistance to DDT in several *Anopheles* species, pyrethroids remain the only insecticides authorized by WHO for extensive use on malaria elimination campaigns [3]. The exploitation of pyrethroids in China started from 1970s, and the application of this new type of insecticide has flourished in recent years. Since 1997, insecticide production in China has been over 3.9×10^8^kg and application around 2.5×10^8^ kg per annum. The area treated with pyrethroids occupies more than one third of the total insecticide-treated area in China [4].

*Anopheles sinensis*, *Anopheles lesteri*, *Anopheles minimus s*.*l*. and *Anopheles dirus s*.*l*. are the four main malaria vectors in China. The most widely distributed and extensively investigated malaria vector is *An*. *sinensis*, and the areas where vector insecticide resistance monitoring has been carried out since the 1990s are located mainly in the eastern and southern provinces. In contrast, there is lack of the systematic, comprehensive and standard monitoring data of malaria vector resistance in recent years, especially from the malaria endemic areas [5].

## Methods

### Study area

**Figure 1:**
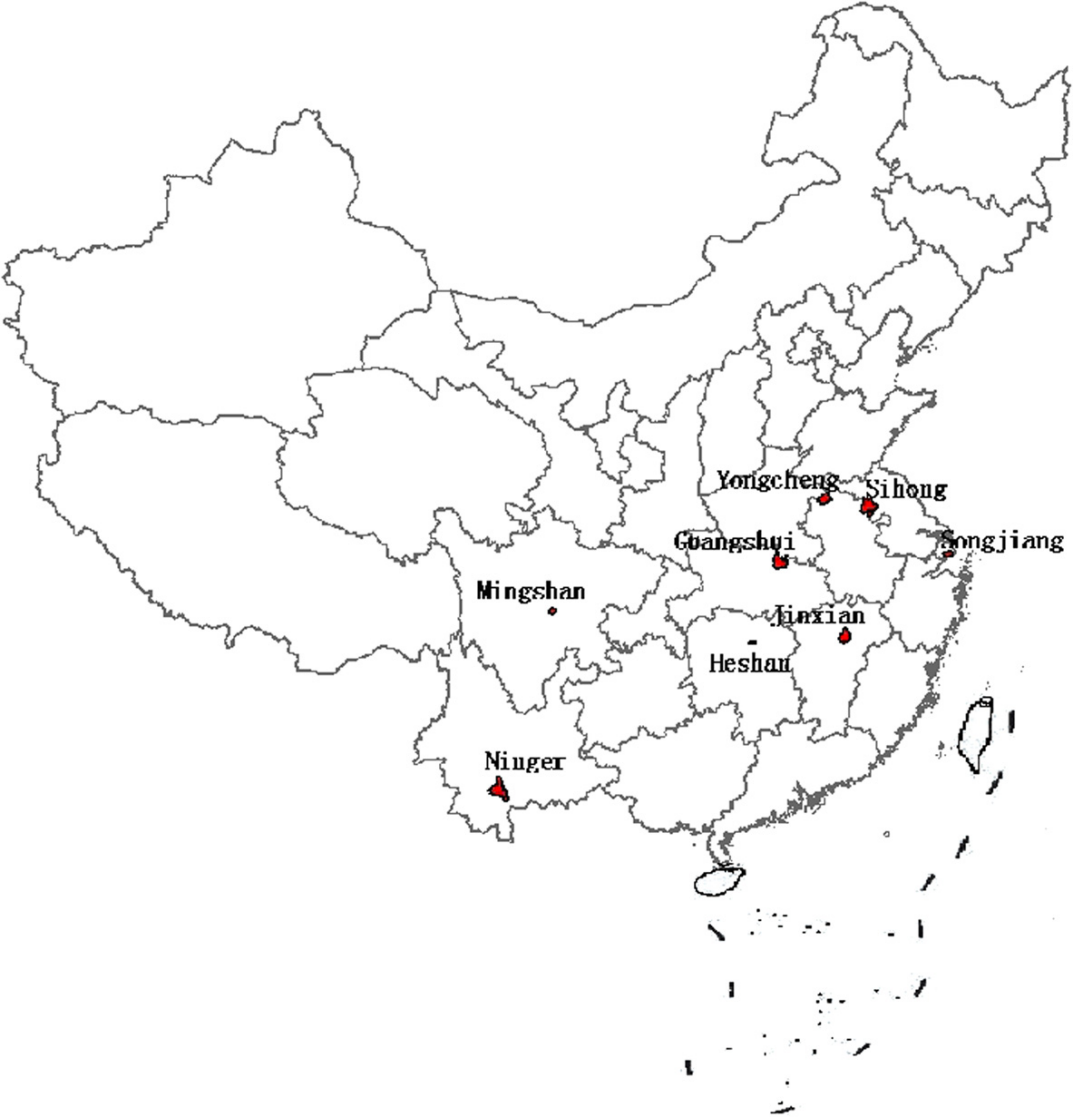
The sentinel sites in this study.

Eight provinces from different malaria endemic regions were selected for study (Figure 1). One county in each province, two or three sentinel sites in each county were chosen for the survey. In the selected county, the sites were chosen to encompass a range of insecticide selection pressures including an urban site with local use of insecticides in subsistence agriculture, a rural site with high coverage of insecticide-based malaria control interventions and a site with less insecticide usage.

### Sampling collection

From August to October in 2010, adult mosquitoes were collected by different collection methods (indoor and outdoor human landing collection, collection on cattle and morning resting collections inside houses) in sentinel sites. Female mosquitoes collected from the field were identified morphologically using taxonomic keys [6]. All samples in each county were pooled and raised together to the F1 families,1-3 day old adult females were used for the insecticide bioassay.

### Resistance tests

Two insecticides were tested: 0.25% deltamethrin and 4% DDT. Insecticide papers were obtained from the WHO reference centre in Malaysia [7]. Each batch was tested on the insecticide susceptible Jiangsu strain of *Anopheles* at the key laboratory in National Institute of Parasitic Diseases, Chinese Center for Disease Control and Prevention before dispatch to the participant provinces. Any batches of papers showing less than 100% mortality in these control tests were discarded. Each paper was used a maximum of six times. About 100 mosquitoes (3–5 replicates of approximately 25 mosquitoes, with tests performed over more than one day) were exposed to each insecticide for 1 hour. Mortality was recorded 24 hours after exposure. Each day a control was tested alongside the exposure tubes. The bioassay result was corrected using the Abbott formula when the control mortality was between 5 and 20%. The bioassay results were summarized in three resistance classes as defined by WHO: (1) susceptible when mortality was 98% or higher, (2) possible resistant when mortality was between 97 and 80%, and (3) resistant when the mortality was lower than 80% [8].

### Ethical considerations

We have obtained ethics approval from National Institute of Parasitic Disease, Chinese Center for Disease Control and Prevention (Who Collaborating Center for Malaria, Schistosomiasis and Filariasis) ethical committee and written informed consent was obtained from all the participants. No specific permissions were required for these activities, the location is not privately-owned and the field studies did not involve endangered or protected species.

## Results

The *An*. *sinensis* reference strain from Jiangsu was susceptible to all insecticides, showing 100% mortality at WHO recommended discriminating dosages. Mortality in control groups was consistently below 5%, and no correction was required.

Exposure to 0.25% deltamethrin of F1 families indicated obvious resistance in eight provinces with mortalities ranging from 5.96% to 64.54% across the study regions. There was a significant difference of mortality; the highest percentage of mosquitoes surviving the WHO diagnostic doses was seen in the agricultural districts of Guangshui County in Hubei Province, while the lowest was recorded of Minshan County in Sichuan Province.

Similarly, a high frequency of DDT resistant individuals was also observed in Hubei Province (9.29% mortality), Jiangxi Province (10.72% mortality) and Hunan Province (23.67% mortality). All the *An*. *sinensis* population collected in the study region showed less than 80% mortality to DDT and was thus defined as resistant at the diagnostic concentration of 4%.

Above all, the results of standard WHO susceptibility tests against adults reared from wild collected in eight provinces during 2010 adults showed *An*. *sinensis* were completely resistant to both deltamethrin and DDT in the study areas (Table 1).

**Table 1:** **WHO insecticide susceptibility test results on 1–3 day old F1 *An. sinensis *reared from females collected from the study area in 2010**

**Province**	**County**	**Deltamethrin (0.25% w/v) **^**a**^			**Resistance status **^**b**^	**DDT (4%w/v) **^**a**^			**Resistance status **^**b**^
		**Total(n)**	**(%) mort**	**95% CI**		**Total(n)**	**(%) mort**	**95% CI**	
Hubei	Guangshui	102	5.96	6.04~5.88	R	97	9.29	9.25~9.33	R
Henan	Yongcheng	108	20.35	20.47~20.23	R				
Hunan	Heshan	107	28.57	28.89~28.25	R	101	23.67	23.52~23.82	R
Jiangsu	Sihong	96	35.72	35.79~35.65	R	106	47.62	47.42~47.82	R
Jiangxi	Jinxian	107	12.35	12.40~12.30	R	102	10.72	10.64~10.80	R
Sichuan	Minshan	92	64.54	57.82~57.18	R				
Shanghai	Songjiang	80	57.5	64.84~64.24	R	120	73.61	73.31~73.91	R
Yunnan	Ninger	100	45.84	45.96~45.72	R	88	52.28	52.22~52.34	R
Susceptible reference strain		107	100.00		S	101	100.00		

Results expressed as % mortality 24 hr post exposure.

^a^WHO diagnostic concentration . ^b^S = susceptible; R = resistant.

## Discussion

### Extensive deltamethrin and DDT resistance in *An*. *sinensis* from malaria-endemic areas which showed a good agreement in mortality rate

**Figure 2:**
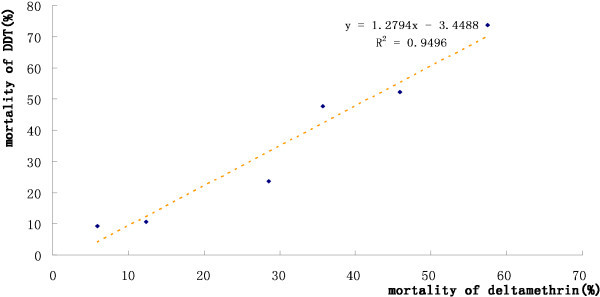
**The relationship between the mortality of DDT and that of deltamethrin in *An. sinensis *population.**

Based on the WHO criteria for characterizing insecticide resistance/susceptibility, the populations of *An*. *sinensis* from the study areas were high resistant to both deltamethrin and DDT, and the most important finding in this study was the good correlation in mortality rate between deltamethrin and DDT at the diagnostic concentration, the lower mortality rate of deltamethrin with lower mortality rate of DDT, which indicated a good agreement between the resistance to deltamethrin and that to DDT in the tested population (Figure 2). This agrees with several recent studies completed in Anhui [9], Hainan [10], Hubei [11] and Jiangsu [12] provinces, where the mortality of *An*. *sinensis* to DDT and deltamethrin were both below 60% at the same diagnostic concentration from 2006 to 2010. It is well known that knockdown resistance (*kdr*) is a type of target-site resistance arising from point mutations in the sodium channel genes of the insect nervous system to confer cross-resistance to DDT and pyrethroids [13]. Similar observations of resistance in populations of *Anopheles arabiensis* to DDT and deltamethrin had also been reported from Sudan [14] and Ethiopia [15]. This study confirms that switching from DDT to deltamethrin can only be a short term strategy, since resistance to both insecticides will increase.

Although malaria mosquitoes have not been treated with DDT for many years in China, obvious resistance to DDT still exists in mosquito populations. For *An*. *sinensis*, resistance to DDT was found to be ubiquitous during 1990s: eighty-nine percent of 27 regions in five provinces exhibited obvious or elementary resistance. The problem in Hubei and Yunnan province was quite striking [5]. Similarly, this present large-scale survey also indicates that DDT resistance still exists in the malaria-endemic areas, while *An*. *sinensis* is completely resistant to DDT in the study sites. The level of DDT resistance has possibly been increased by the agricultural and domestic use of deltamethrin [16], through the sodium channel genes [13] as well as its resistance can be inherited [5].

For *An*. *sinensis*, deltamethrin and permethrin are the major pyrethroid insecticides, and the monitoring data recorded during 1990s in China showed elementary resistance or sensitivity to these two insecticides in most of the regions investigated [5], and the occurrence of initial resistance during the 2000s has been reported in some areas, while obvious resistance was ubiquitous of in recent years [17-22]. The resistance to deltamethrin has risen significantly in the malaria-endemic areas compared to those in the 1990s.

Moreover, almost all studies had been accomplished during 1990s using the modified criteria for Chinese case which were far below [21] that initiated and standardized by the WHO in the mid-1980s. Since 2000, most related studies have been completed using WHO standard at the same time. When the diagnostic concentration was considered, the resistance to deltamethrin has risen more seriously than the collected data indicating.

Since pyrethroids have been extensively used in recent years, the area treated with pyrethroids occupies more than one third of the total insecticide-treated area in China. For the public health market, pyrethroids are used as indoor sprays or incense, or to impregnate bed nets, curtains and screens. Many highly efficient, non-toxic pyrethroids are applied extensively in agriculture and public sanitation, such as deltamethrin, permethrin, methothrin, resmethrin and cyhalothrin.

Therefore, it is not surprising that *An*. *sinensis* resistance to deltamethrin has risen quickly from 1990–2010, and this large-scale survey further indicated that pyrethroids resistance in *An*. *sinensis* was developing quickly during these twenty years, though the surveys covered eight provinces that vary substantially in geography, economic and social environmental changes, there was unanimous agreement among the result that high level of resistance against the two insecticides existing in all survey sites with most of the population showing mortality lower than 65%. Excessive use of agricultural pesticides may be blamed for causing such insecticide resistance increase during these years.

Since pyrethroid resistance mainly results from agricultural applications, it is likely that such resistance will evolve regardless of the organized use of pyrethroids in properly managed malaria control campaigns. Even if pyrethroid resistance is a serious threat to vector control, it would be dangerous to extrapolate from these results alone before the geographical extent of the resistance and its impact on the protective effect of impregnated bed nets has been determined.

Vector control is an essential component of malaria control programmes. The WHO has reaffirmed the importance of vector control through indoor residual spraying (IRS) as one of the primary interventions for reducing or interrupting malaria transmission in both stable and unstable transmission zones. Though resistance does not necessarily result in failure to control disease, sufficient attention should be paid to pyrethroid resistance in malaria vectors either from endemic or no endemic areas in China, since the integrated approach to vector control is one of the key integral components for its malaria elimination programme. Even an integrated approach to vector control has frequently been advocated [23-25]. Although the need for a reduced reliance on insecticides for vector-borne disease control has been stressed further by the Intergovernmental Forum on Chemical Safety, Forum VI [26], in fact various studies predicted that combinations of interventions can be much more effective in reducing malaria transmission than individual interventions and that the effect of IRS and ITNs is amplified by environmental management, even in areas of intense transmission [27,28]. Besides its direct effect on transmission intensity, the integration of methods may also contribute to resistance management. For example, larval control is expected to prevent or delay the onset of vector resistance to insecticides [29], whereas measures that reduce human contact with vectors, such as good housing conditions or presence of repellents, will reduce the selection pressure.

Operationally in China, it is urgent to set up networks to systematically and continuously monitor the resistance levels and follow their evolution among major *Anopheles* mosquitoes to main insecticides, especially in areas where vector control operations are planned for the malaria elimination programme. It is also important to strengthen basic and operational research to clarify the resistance mechanisms and evaluate the impact of resistance on the efficacy of insecticide-based vector control measures in the field, so that rational insecticide choice can be made and the successful contribution of vector control to malaria elimination could be ensured.

## Competing interests

The authors hereby certify that no conflict of interest of any kind occurred in the framework of this study.

## Authors’ contributions

XZG organized and supervised the field work, WDQ analysed the data and completed the manuscript, ZXN and ZSS provided the administrative support, WRB and ZQF helped in the study design and reviewed the manuscript. All authors read and approved the final manuscript.
